# 2-Hydroxy Arachidonic Acid: A New Non-Steroidal Anti-Inflammatory Drug

**DOI:** 10.1371/journal.pone.0072052

**Published:** 2013-08-27

**Authors:** Daniel H. Lopez, Maria A. Fiol-deRoque, Maria A. Noguera-Salvà, Silvia Terés, Federica Campana, Stefano Piotto, José A. Castro, Raheem J. Mohaibes, Pablo V. Escribá, Xavier Busquets

**Affiliations:** 1 Lipopharma Therapeutics, Palma de Mallorca, Balearic Islands, Spain; 2 Laboratory of Cell Biology, Department of Biology-IUNICS, University of the Balearic Islands, Palma de Mallorca, Balearic Islands, Spain; 3 Department of Pharmaceutical and Biomedical Sciences, University of Salerno, Fischiano, Salerno, Italy; 4 Laboratory of Genetics, Department of Biology-IUNICS, University of the Balearic Islands, Palma de Mallorca, Balearic Islands, Spain; 5 Laboratory of Molecular and Cellular Biomedicine, Department of Biology-IUNICS, University of the Balearic Islands, Palma de Mallorca, Balearic Islands, Spain; University of Navarra School of Medicine and Center for Applied Medical Research (CIMA), Spain

## Abstract

**Background:**

Nonsteroidal anti-inflammatory drugs (NSAIDs) are a family of COX1 and COX2 inhibitors used to reduce the synthesis of pro-inflammatory mediators. In addition, inflammation often leads to a harmful generation of nitric oxide. Efforts are being done in discovering safer NSAIDs molecules capable of inhibiting the synthesis of pro-inflammatory lipid mediators and nitric oxide to reduce the side effects associated with long term therapies.

**Methodology/Principal Findings:**

The analogue of arachidonic acid (AA), 2-hydroxy-arachidonic acid (2OAA), was designed to inhibit the activities of COX1 and COX2 and it was predicted to have similar binding energies as AA for the catalytic sites of COX1 and COX2. The interaction of AA and 2OAA with COX1 and COX2 was investigated calculating the free energy of binding and the Fukui function. Toxicity was determined in mouse microglial BV-2 cells. COX1 and COX2 (PGH2 production) activities were measured in vitro. COX1 and COX2 expression in human macrophage-like U937 cells were carried out by Western blot, immunocytochemistry and RT-PCR analysis. NO production (Griess method) and iNOS (Western blot) were determined in mouse microglial BV-2 cells. The comparative efficacy of 2OAA, ibuprofen and cortisone in lowering TNF-α serum levels was determined in C57BL6/J mice challenged with LPS. We show that the presence of the –OH group reduces the likelihood of 2OAA being subjected to H* abstraction in COX, without altering significantly the free energy of binding. The 2OAA inhibited COX1 and COX2 activities and the expression of COX2 in human U937 derived macrophages challenged with LPS. In addition, 2OAA inhibited iNOS expression and the production of NO in BV-2 microglial cells. Finally, oral administration of 2OAA decreased the plasma TNF-α levels in vivo.

**Conclusion/Significance:**

These findings demonstrate the potential of 2OAA as a NSAID.

## Introduction

Chemical modification of fatty acids is an experimental approach used to inhibit cyclooxygenase 1 (COX1) and cyclooxygenase 2 (COX2) activity [1]. We rationally designed and synthesized 2-hydroxy-arachidonic acid (2OAA), which contains a hydroxyl group on the α-carbon of arachidonic acid (AA), a modification that was designed to inhibit the AA pro-inflammatory pathway by interacting with the active site of COX1 and COX2. AA is the most abundant n-6 polyunsaturated fatty acid found in the cell membrane [2], where it is stored. When phospholipase A2 (PLA2) is activated by different inflammatory stimuli, including bacterial lipopolysaccharides (LPS), cytokines and allergens [3], [4], AA is released into the cytosol and then metabolized by cyclooxygenases (COXs), lipoxygenases (LOXs) and cytochrome P450 [2]. Two major COXs isoforms have been described, the constitutive (COX1) and the inducible (COX2) [5]. When metabolized by COX1 and COX2, AA is converted by a variety of downstream enzymes (isomerases, reductases and synthases), including the prostaglandins (PGs) and thromboxanes (TXs). The LOX pathway metabolizes the AA to hydroxyacids and leukotrienes and the P450 pathway to epoxyeicosatrienoic acids or 20-hydroxyeicosatetraenoic acid. Finally the AA-derived bioactive products are released from activated cells to modulate the inflammatory response [6], [7].

An additional key process in inflammation is the synthesis of nitric oxide (NO) by one of the three nitric oxide synthase (NOS) isoenzymes: the constitutive, neuronal and inducible (iNOS) isoforms [8]. The iNOS isoform is upregulated by a variety of proinflammatory stimuli and it mediates pathogen killing, vasodilatation and vascular permeability [9]. Moreover, nitric oxide is also oxidized and converted to peroxinitrite, which exerts a variety of cytotoxic effects [9]. Acute and chronic inflammatory responses induced by AA metabolites and NOS activity are associated with important pathological processes, such as rheumatoid arthritis [10], asthma [11], cystic fibrosis [12], cancer [13] and Alzheimer's disease [14].

Here, we demonstrate that the 2-hydroxy-modified form of AA, 2OAA, inhibits COX1 and COX2 activity, as well as COX2 expression in macrophages. Moreover, it diminishes iNOS expression and NO production in microglia cells, and it decreases tumor necrosis factor alpha (TNF-α) levels *in vivo* when C57BL6/J mice are challenged with LPS. Taken together, the ease of administration (orally), and strong efficacy of 2OAA suggest that this compound potentially constitutes a useful anti-inflammatory drug.

## Materials and Methods

### Ethics Statement

This study was carried out in strict accordance with National Legislation (Real decreto 1201 2005 according to European law 86609/CE (UE).

The protocol was approved by the Bioethics Committee of the University of Balearic Islands (Permit Number: 2622007, 6352007). All efforts were made to minimize suffering.

### Binding energy study: molecular docking simulations

The preparation of the COX1 (pdb entry 2OYE) and COX2 (pdb entry 1DCX) structure was performed as described previously [15], using the final conformation of COX2 to reorient the COX1 structure. This operation permitted the binding box to be superimposed and easy automatization of the docking screening. Molecular dynamics calculations were carried out using Yasara software [16], using a simulation box of 107.11 Å, 75.44 Å, and 85.73 Å for the a, b, and c axes, respectively, under periodic boundary conditions. Simulations were carried out under the NPT ensemble at 310K and 1 atm by coupling the system to a Berendsen thermostat and controlling the pressure in the manometer pressure control mode [17].

The AMBER03 force field [18] was used to calculate the binding energies for the R and S 2OAA enantiomers, and force field parameters were generated using the Autosmiles method [19]. Briefly, the geometry of monomers was optimized using the semi-empirical AM1 method and the COSMO solvation model. Partial atomic charges were calculated using the same theory level as in the Mulliken point charge approach, and they were then improved by applying the AM1 Bond Charge Correction.

Electrostatic interactions were calculated with a cut-off of 7.86 Å, and long-range electrostatic interactions were handled with the Particle Mesh Ewald algorithm using a sixth-order B-spline interpolation and a grid spacing of 1 Å. After removal of the conformational stress by short steepest descent minimization, the procedure was continued by simulated annealing (time step 1 fs, atom velocities scaled down by 0.9 every 10^th^ step) until convergence was reached (*i.e.*, the energy improved by less than 0.0002 kcal/mol per atom over 200 steps).

To determine the flexibility of COX receptors, we ran docking experiments on the 10 structures obtained by sampling every 1 ns with dynamics of 10 ns. Binding energies were calculated using the Autodock 4 program [20] with the AMBER03 force field.

Measurement of the binding energy is insufficient to evaluate the potential inhibitory activity of 2OAA and it is also important to estimate the energy required to extract a hydrogen from the carbon C13 in AA and 2OAA. One of the most effective and best known methods to assess the reactivity of a chemical species *a priori* is the frontier orbital theory of Fukui, further developed by Parr and Yang [21]. This method relates the reactivity of a molecule, with respect to electrophilic, nucleophilic, and radical attack, to the charge density. These so-called Fukui functions provide a qualitative means of measuring and displaying the reactivity of regions of a molecule. Specifically, the f^0^(r) measures the sensitivity of the charge density, ρ(r), to the loss or gain of electrons via the following equation:
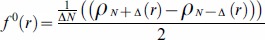



A molecule is susceptible to radical attack at sites where f^0^(r) is large. The calculation was performed using the DMol3 program [22] with functional pwc, TNP basis set, Fermi occupation, and a global cutoff of 3 Å. The grid resolution was set to 0.15 Å.

### Cell culture

The human monocyte U937 cell line (ATCC® CRL-593.2™) was kindly provided by Dr Amanda Iglesias (Hospital Universitario Son Dureta, Balearic Islands, Spain). These cells were cultured at 37°C in RPMI 1640 medium supplemented with 10% FBS (Fetal Bovine Serum), HEPES (10 mM), penicillin (100 units/ml) and streptomycin (0.1 mg/ml), and in a humidified atmosphere with 5% CO_2_. To differentiate U937 cells to a macrophage-like phenotype, they were plated onto 6-well collagen coated plates at a density of 8×10^5^ cells/well in the presence of Phorbol Myristate Acetate (PMA; 100 nM) diluted in dimethyl sulfoxide (DMSO) and incubated for 72 h. DMSO was always present at a final concentration of 0.1%.

Differentiated macrophage-like cells were characterized by their attachment to the collagen-coated plates, the presence of filopodia and the expression of the intercellular adhesion molecule 1, as determined in Western blots (data not shown). Once differentiated, the culture medium was removed and replaced with fresh RPMI 1640 medium containing 5% FBS, HEPES (10 mM), penicillin (100 units/ml) and streptomycin (0.1 mg/ml).

BV-2 cells, Inter Lab Cell Line Collection (ICLC code: ATL03001) were obtained from the Istituto Nazionale Ricerca sul Cancro (Genova, Italy) and they were grown at 37°C in RPMI 1640 medium supplemented with 10% FBS, penicillin (100 units/ml) and streptomycin (0.1 mg/ml), and in a humidified atmosphere with 5% CO_2_.

### Cell viability assays

Cell proliferation in the absence and presence of 2OAA were determined using the methylthiazolyl diphenyl tetrazolium bromide (MTT) method [23] and the Trypan blue staining method [24]. 2OAA was sinthetized and purchased from Lipopharma Therapeutics (Mallorca, Spain).

MTT: BV-2 cells were plated in 96-well plates at a density of 3×10^3^ cells/well and with 150 µl culture medium (10% FBS) per well. After incubating overnight to allow cell attachment, cells were treated with 30×10^−6^–240×10^−6^ M of 2OAA or AA for 24 h, after which 10% MTT (5 mg/ml in PBS 1X) reagent was added and the cells were incubated for a further 4 h. The medium was removed and 200 µl of DMSO was added to the cells and the plates gently shaken. Absorbance at 550 nm was measured 5 min later using a micro plate reader (Asys Hitech, Eugendorf, Austria).

Trypan Blue staining: BV-2 cells were plated in 6-well plates at densities of 3×10^5^ cells/well with 2 ml of culture medium (10% FBS) per well. After incubating overnight to allow cell attachment, cells were treated with 30×10^−6^–240×10^−6^ M of 2OAA or AA for 24 h, Trypan blue staining was done as previously described [24]. Briefly, 10 µl of sample (cell suspension) was mixed with 10 µl of trypan blue (Invitrogen), and pipetted into a Countess® chamber slide (Invitrogen) that was inserted in the Countess® Automated Cell Counter (Invitrogen).

### Assays of COX1 and COX2 inhibition *in vitro*

The effect of 2OAA and AA was assessed on purified COX1 and purified COX2 proteins using an *in vitro* cell free-system inhibitor assay kit provided by Cayman Chemicals (Ann Arbor, MI, USA). in accordance with the manufacturer directions. Both assays quantifyed the levels of prostaglandin H_2_ (PGH_2_) produced by purified COX1 or purified COX2 [25]. The results were expressed as the percentage of COX1 or COX2 activity in the presence or absence (control 100%) of 2OAA (250×10^−6^ M) or AA (250×10^−6^ M).

### Western blotting and protein quantification

To quantify COX1 and COX2 protein, U937 cells were differentiated in 6-well plates at a density of 8×10^5^ cells/well, and incubated for 24 h in culture medium containing 5% FBS (6 ml/well). Next, the culture medium was removed and replaced with fresh medium containing 2OAA (120×10^−6^ M) for 1 h or incubated with fresh culture medium alone (control). The cells were then washed twice with PBS and the cells previously treated with 2OAA were challenged for 6 h with LPS (62 ng/ml) plus 2OAA (120×10^−6^ M) in fresh medium, while untreated control cells were challenged with LPS alone (62 ng/ml).

To study COX2 protein decay, U937 cells were differentiated in 6-well plates as above and after removing the culture medium, the cells were treated for 6 h with LPS (62 ng/ml) plus 2OAA (120×10^−6^ M). After washing twice with PBS, the cells were then treated for 2 h with cycloheximide (CHX, 50×10^−6^ M) and NS-398 (20×10^−6^ M, a COX2-specific inhibitor) or MG-132 (20×10^−6^ M, a 26 S proteasome inhibitor) in the presence of 2OAA (120×10^−6^ M).

To quantify the iNOS protein, BV-2 cells were plated in 6-well plates as described above, and the culture medium was then removed and replaced with fresh medium containing 2OAA (120×10^−6^ M) for 1 h (or with fresh culture medium alone, control). The cells were then washed twice with PBS and 2OAA-treated cells were challenged for 24 h with LPS (1 µg/ml) plus 2OAA (120×10^−6^ M) dissolved in fresh medium. Untreated control cells were incubated for 24 h in the presence or absence of LPS (1 µg/ml) dissolved in fresh medium.

At the end of the treatments, both U937 and BV-2 cells were washed twice with PBS and harvested with a rubber policeman in 200 µl of protein extraction buffer (10 mM Tris-HCl buffer [pH 7.4], 50 mM NaCl, 1 mM MgCl_2_, 2 mM EDTA, 1% SDS, 5 mM iodoacetamide and 1 mM PMSF). The cell suspensions were sonicated for 10 s at 50 W using a Braun Labsonic U sonicator and 20 µl aliquots were removed for protein quantification (bicinchoninic acid method; Pierce-Thermo Fisher Scientific Inc., Roskilde, Denmark). The remaining suspension (∼180 µl) was mixed with 20 µl of 10× electrophoresis loading buffer (120 mM Tris-HCl [pH 6.8], 4% SDS, 50% glycerol, 0.1% bromophenol blue, 10% β-mercaptoethanol) and boiled for 5 min. The proteins were fractionated on 9.5% polyacrylamide gels (SDS-PAGE: 15-well and 1.5 mm thick) and transferred to nitrocellulose membranes (Whatman protran®, Dassel, Germany) that were then blocked for 1 h at room temperature in PBS containing 5% non-fat dry milk, 0.5% bovine serum albumin and 0.1% Tween 20 (blocking solution). The membranes were probed overnight at 4°C with one of the following primary antibodies diluted in blocking solution: monoclonal anti-COX1/anti-COX2 (1∶800; Biotechnology Inc, CA, USA), anti-iNOS (1∶4,000; BD Biosciences, Franklin Lakes, NJ, USA), or anti-α-tubulin (1∶10,000; Sigma-Aldrich, St. Louis, MO, USA).

After removing the primary antibody, the membranes were washed three times for 10 min with PBS and incubated for 1 h at room temperature in fresh blocking solution containing horseradish peroxidase-linked goat anti-mouse IgG (1∶2000; Amersham Pharmacia). Immunoreactivity was detected by Enhanced Chemiluminescence (ECL; Western Blot Detection system, Amersham Pharmacia), followed by exposure to ECL hyperfilm (Amersham Pharmacia). The films were scanned at 600 dpi and quantified using Foto Look 32 software (Agfa Gevaert, Leverkusen, Germany), analyzing the images with Quantity One software (Bio-Rad, Hercules, CA, USA). The concentration of a given protein was normalized to the α-tubulin content of the same sample.

### Quantitative Real Time-Polymerase Chain Reaction (qRT-PCR) and mRNA quantification

To quantify COX2 mRNA, U937 cells were differentiated in 6-well plates as described above and the culture medium was then replaced with fresh medium containing 2OAA (120×10^−6^ M) for 1 h, or incubated with fresh culture medium alone (control). The cells were washed twice with PBS, and those previously treated with 2OAA were challenged for 6 h with LPS (62 ng/ml) plus 2OAA (120×10^−6^ M) in fresh medium, while control cells were challenged for 6 h with LPS alone (62 ng/ml). Total cellular RNA was extracted from the cells using the RNeasy Mini kit in combination with the RNase-Free DNase kit (Qiagen, Hilden Germany), following the manufacturer's instructions, and the total amount and purity of RNA was measured using a Nanodrop 1000 spectrophotometer (ThermoFisher Scientific, Waltham, MA) at 260 and 280 nm. The integrity of the RNA was tested by electrophoresis on 2% agarose gel and visualized by ethidium bromide staining. Reverse transcription was carried out in a final volume of 20 µl using the Transcriptor First Strand cDNA Synthesis Kit (Roche, Mannheim, Germany) in a thermal cycler (Eppendorf Master Cycler Gradient). The RNA samples (1 µg) were mixed with oligonucleotides as primers (1 µl; 500 µg/ml) and made up to a final volume of 13 µl with H_2_O. The samples were then incubated at 65°C for 10 min and then the tubes were chilled quickly on ice. Next, a reaction mix was added containing 5× first-strand buffer (4 µl), 10 mM dNTPmix (dGTP, dCTP, dATP, and dTTP; 2.5 µl), Protector Rnase inhibitor (0.5 µl; 40 units/µl) and the reverse transcriptase (0.5 µl; 20 units/µl). The reaction mixtures were incubated at 25°C for 10 min, 55°C for 30 min, and 85°C for 5 min, and the cDNA samples obtained were stored at −20°C.

For PCR amplification, primers were designed based on specific human COX2 sequences obtained from the SDSC Biology Workbench Program: forward primer, 5′-TGA GCA TCT ACG GTT TGC TG-3; reverse primer, 5′- TGC TTG TCT GGA ACA ACT GC-3′. As an endogenous control, the expression of β-actin was quantified in parallel using the forward primer 5′-GCG GGA AAT CGT GCG TGA CAT T-3′ and the reverse primer 5-CTA CCT CAA CTT CCA TCA AAG CAC-3′. RT-qPCR amplifications were carried out in a Step One Plus v 2.0 thermal cycler (Applied Biosystems) using the SYBR® Premix Ex Taq kit (Perfect Real Time, Takara), which contains Ta*K*aRa Ex Taq™ HS, dNTP mixture, Mg^2+^, SYBR®, Green I and ROX™ Reference Dye. Thermal cycling was preceded by an initial denaturation step at 95°C for 30 s. and DNA amplification and fluorescence quantification was performed over 35 cycles with a denaturation step at 95°C for 5 s, followed by an annealing and extension step at 60°C for 34 s. Fluorescence quantification was carried out after each DNA extension step, and StepOne software (v2.0) was used to analyze the data, also producing a melting curve analysis of the final products.

The ratio of COX2 expression to that of β-actin RNA (whose expression is not modulated by 2OAA) was expressed as ddCT values (as a percentage) using the following formula: ddCT = EX ^(Ctc-Ctx)^/E Bact ^(Ctc-Ctx)^, where Efficiency (E) = 10 (−1/m), and (m) = slope of the graph formed by Ct values of mRNA vs the logarithm (log) of its concentration (ng/µl). Finally, this value was used to calculate the relative expression of COX2 in 2OAA-treated cells with respect to the untreated control cells.

### COX2 immunofluorescence detection

U937 cells were differentiated for 24 h in 8-well collagen-coated plates containing 750 µl of culture medium (5% FBS) at a density of 9×10^4^ cells/well. After incubating in the presence or absence (control) of 2OAA (120×10^−6^ M) for 1 h, the cells were washed twice with PBS and 2OAA-treated cells were challenged with LPS (62 ng/ml) plus 2OAA (120×10^−6^ M) for 18 h, while control cells were incubated for 18 h in the presence or absence of LPS alone (62 ng/ml).

The cells were washed twice with PBS, twice with phosphate buffer (PB; 0.1 M), and fixed with 4% paraformaldehyde for 30 min. Cells were then washed once with PB (0.1 M), twice with PBS, and permeabilized with Triton X-100 (0.1%) for 5 min. Subsequently the cells were washed with PBS three times and incubated with 10% FBS in PBS for 3 h at room temperature, followed by overnight incubation at 4°C in the presence of monoclonal anti-COX2 (1∶50; Biotechnology, INC, CA, USA) diluted in PBS buffer supplemented with 10% FBS. The cells were then washed three times with PBS and incubated for 1 h at room temperature with Alexa Fluor 488-labelled goat anti-mouse IgG antibody (1∶200; Invitrogen, Molecular Probes; excitation at 488 nm and emission at 510–550 nm). Finally, cells were washed twice with PBS, incubated with 500 nM propidium iodide for 4 min to stain the nuclei and washed once again with PBS. Images were acquired using a Leica TCS SP2 spectral confocal microscope, with 630× optical magnification and 8× digital magnification (∼5000× total magnification).

### Nitric oxide determination

BV-2 cells were plated in 96-well plates at a density of 2×10^4^ cells/well in 200 µl of culture medium (10% FBS) and after 24 h, the culture medium was replaced with fresh medium alone (control) or containing 2OAA (50×10^−6^, 120×10^−6^ and 240×10^−6^ M). After 1 h, the cells were washed twice with PBS and 2OAA-treated cells were challenged for 24 h with LPS (1 µg/ml) plus 2OAA (120×10^−6^ M), while control cells were incubated for 24 h in the presence or absence of LPS (1 µg/ml) alone. The NO produced by the cells was determined by assaying the amount of nitrite generated using Griess reagent [26]. Briefly, aliquots of the conditioned medium were mixed with an equal volume of 1% sulfanilamide in 5% phosphoric acid and 0.1% N-1-naphthylenediamine-dihydrochloride in water. After incubating for 10 min at room temperature, the absorbance of the reaction mixture at 540 nm was determined in a microtiter plate reader, using sodium nitrite diluted in culture medium at concentrations of 1.5–25 µM to generate a standard curve.

### Determination of TNF-α serum levels

We measured the effect of oral 2OAA administration on TNF-α serum levels in a model of transient endotoxemia: C57BL6/J mice (Charles River, Paris, France) challenged with LPS. The protocol used in this study was approved by the Animal Ethics Committee of the University of the Balearic Islands. C57BL6/J mice (15–16 g; 6–8 weeks old) were fed a standard laboratory diet with *ad libitum* access to water and they were maintained on a 12-h light-dark cycle at 22°C. The mice were randomly distributed into 5 groups of 5 animals, of which three groups were administered 2OAA dissolved in PBS (50, 200 or 500 mg/kg, p.o.), while the remaining two groups received PBS (vehicle) alone (control). Doses of 2OAA in mice were calculated following the guidelines of the Food and Drug Administration of the U.S.A (FDA) [27]. After 90 min, the mice were challenged with LPS dissolved in PBS (20 µg, i.p.), while one of the control groups received an i.p. injection of PBS alone. After establishing the optimal dose of 2OAA required to decrease TNF-α serum level, we compared its efficacy with that of cortisone and ibuprofen. Four groups of mice (5 mice per group) were treated with 2OAA (500 mg/kg, p.o.), AA (500 mg/kg, p.o.), or with therapeutic doses of oral ibuprofen (12.5 mg/kg, p.o.) or cortisone (7.5 mg/kg, i.p.), while another two groups (5 mice per group) received PBS alone (control groups). After 90 min the mice were challenged with LPS in PBS (20 µg, i.p.), while one of the control groups received an i.p. injection of PBS alone. Three hours later the mice were sacrificed by decapitation, their blood was collected and the serum separated by centrifugation, and the TNF-α levels were determined by ELISA (Invitrogen, Barcelona, Spain) according to the manufacturer's instructions.

### Statistical analysis

The results are expressed as the mean ± SEM values of at least three independent experiments. Statistical analyses were performed using the Student's *t* test (GraphPad Prism version 5.00 for Windows) and the level of statistical significance was set at *p*<0.05.

## Results

### Computational simulations based on molecular docking

**Figure 1:**
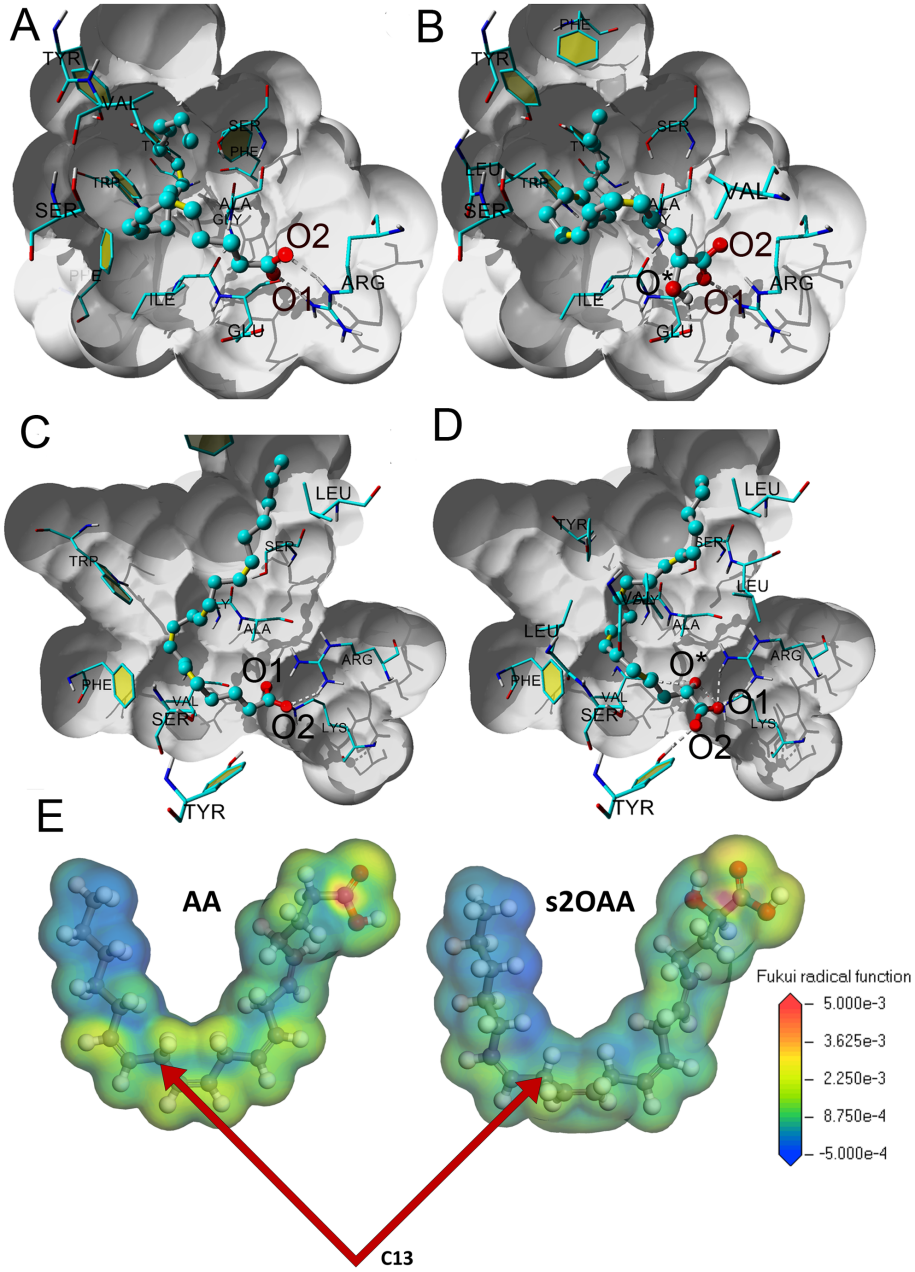
Computational simulations based on molecular docking. **A**. AA in the COX1 binding site. **B**. S2OAA in the COX1 binding site. The two carboxyl oxygens (O1 and O2) of AA establish hydrogen bonds with Arg 120 and they have close hydrophobic contacts with Phe 205, Val 344 and Tyr 348. The orientation of S2OAA is very similar to that of AA, with O1 and O2 occupying the same positions in both. The hydroxyl oxygen (O*) of S2OAA is hydrogen bound to Glu 524, although this favorable interaction is counterbalanced by a distortion of the carbon backbone. To facilitate visual inspection of the interactions, the binding site is shaded in grey, the fatty acids are represented by sticks and balls, and only amino acids closer than 3 Å are shown. **C**. AA in the COX2 binding site. **D**. S2OAA in the COX2 binding site. The carboxylate group of AA is coordinated with Arg 2120 by one hydrogen bond, whereas R2OAA possesses five hydrogen bonds. The O* oxygen occupies the position of O1 of AA and in an analogous manner, O1 of S2OAA occupies the position of the AA O2. Finally, O2 of substituted arachidonic acid is free to hydrogen bond to Tyr 2355. The binding site is shaded in grey, fatty acids are represented by sticks and balls, and only amino acids closer than 3 Å are shown. **E**. The Fukui function f^0^(r) is color-mapped onto the electron density isosurface with equal isovalues.

We compared the binding energy of AA and 2OAA to COX1 and COX2 using computational simulations based on molecular docking. For COX1, the binding energies of AA and 2OAA enantiomers (R2OAA and S2OAA) were very similar (Table 1). The two carboxyl oxygens (O1 and O2) of AA established hydrogen bonds with Arg 120, and AA exhibited close hydrophobic contact with Phe 205, Val 344 and Tyr 348 (Fig. 1A). The orientation of S2OAA (as well as R2OAA, which is not represented in the figure) was very similar to that in AA, with the oxygens O1 and O2 occupying the same positions (Fig. 1B). The hydroxyl oxygen (O*) of R2OAA and S2OAA formed a hydrogen bond with Glu 524 of COX1, although this favorable interaction was counterbalanced by a distortion of the carbon backbone (the RMSD between AA and S2OAA was 1.39 Å). The S enantiomer exhibited a better global interaction with the binding site and the binding energy was slightly higher than that of AA. In summary, the carboxyl groups of the inhibitors were essentially superimposable, and only modest differences in the binding of AA, R2OAA and S2OAA were detected (Table 1).

**Table 1:** Binding energy to COX isoforms.

Receptor	Ligand	Binding energy (kcal/mol)
**COX1**	AA	8.29
	R2OAA	7.94
	S2OAA	8.52
**COX2**	AA	10.25
	R2OAA	11.09
	S2OAA	10.93

For COX2, the binding energies of the 2OAA enantiomers, R2OAA (11.09 kcal/mol) and S2OAA (10.93 kcal/mol), were higher than that of AA (10.25 kcal/mol: Table 1), with a higher degree of hydrogen bonding between 2OAA and the receptor. The carboxyl group of AA was coordinated with Arg 120 via one hydrogen bond (Fig. 1C), while both R2OAA and S2OAA exhibited 5 hydrogen bonds, two of which were severely distorted (Fig. 1D).

Interestingly, in COX2 the O* oxygen of 2OAA occupied the position of O1 of AA (Fig. 1C and 1D), while in an analogous manner, O1 of R2OAA and S2OAA occupied the position of the AA O2. Finally, O2 of R2OAA and S2OAA was free to form a hydrogen bond with Tyr 355.

The map of the Fukui radical function for the total electron density (Fig. 1E) clearly indicates the more favorable sites for radical attack and the arrows highlight C13, the carbon involved in H* abstraction in COX. Thus, it can be thus concluded that the –OH group in C2 reduces the likelihood of 2OAA being subjected to enzymatic alteration by COX isozymes.

### Toxicity

**Figure 2:**
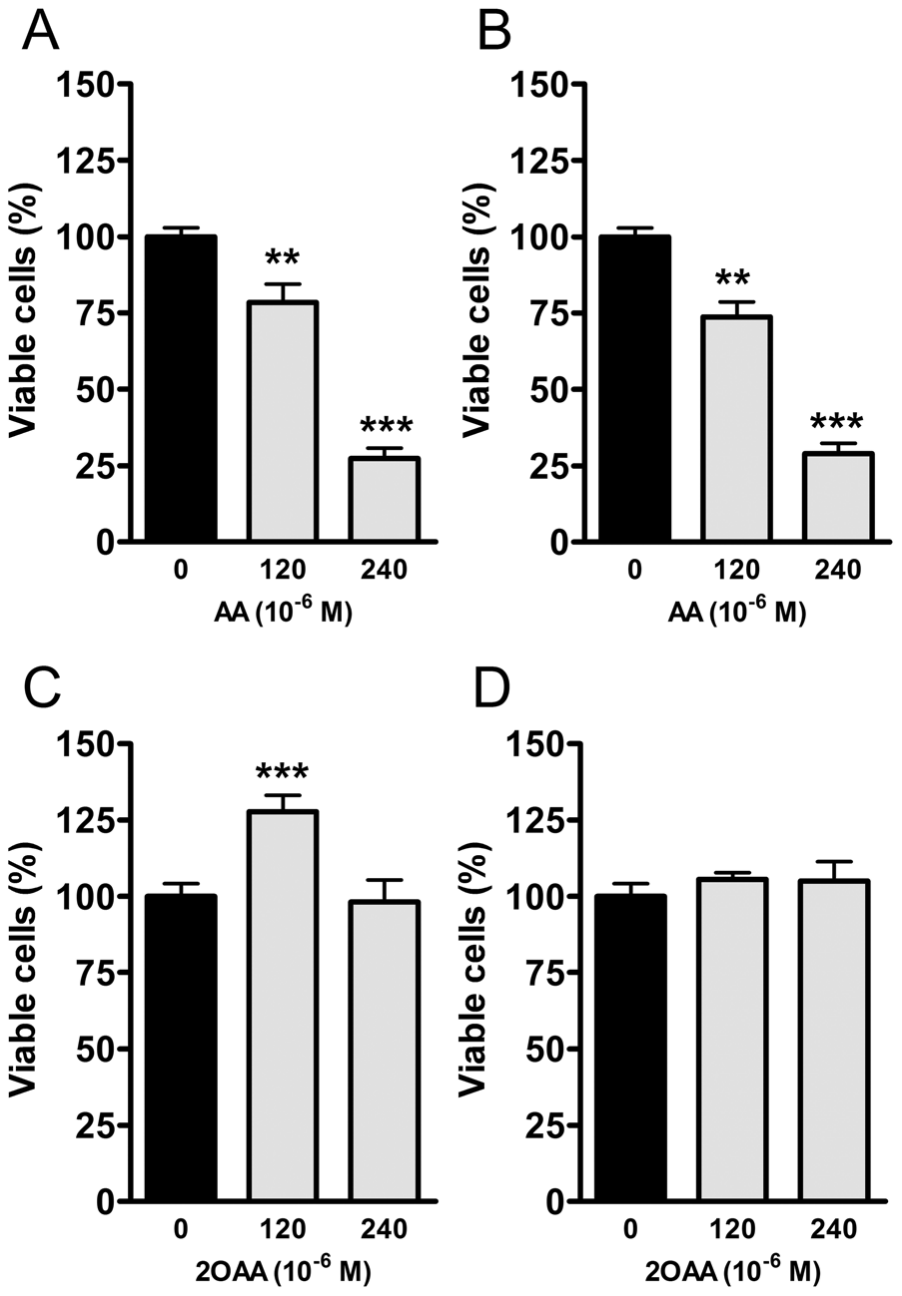
BV2 cell viability is inhibited by AA but not by 2OAA. BV2 mouse cells were exposed to 120 and 240×10^−6^ M of AA or 2OAA for 24 h. Upper panel. Bar diagram showing the cell viability assayed with **A**. MTT or **B** Trypan Blue by AA (120 and 240×10^−6^ M) as compared with untreated control cells (100%; n = 6). Lower panel. Bar diagram showing no inhibitory effect on cell viability assayed by **C** MTT or **D** Typan Blue by 2OAA (120 and 240×10^−6^ M) as compared with untreated control cells (100%: ** p<0.01, *** p<0.001; n = 6).

To determine the toxicity of AA and 2OAA, we measured the effect of both compounds on BV-2 microglial cell viability by the MTT assay and by the Trypan blue staining method. Cells were incubated for 24 h in the presence or absence (control) of AA or 2OAA (120×10^−6^ or 240×10^−6^ M). BV-2 microglial cell viability was inhibited by AA, reaching ∼25% (MTT, Fig. 2A) and ∼24% (Trypan blue, Fig. 2B) inhibition at 120×10^−6^ M and ∼75% (MTT, Fig. 2A) and ∼72% (Trypan blue, Fig. 2B) inhibition at 240×10^−6^ M. By contrast, 2OAA had no negative effect on cell viability at the same concentrations (Fig. 2C and 2D), indicating that the hydroxyl group present in 2OAA attenuates the toxicity exerted by the natural fatty acid AA and suggesting that 2OAA has no toxic effects at therapeutic doses.

### 2OAA inhibits COX1 and COX2 activity

**Figure 3:**
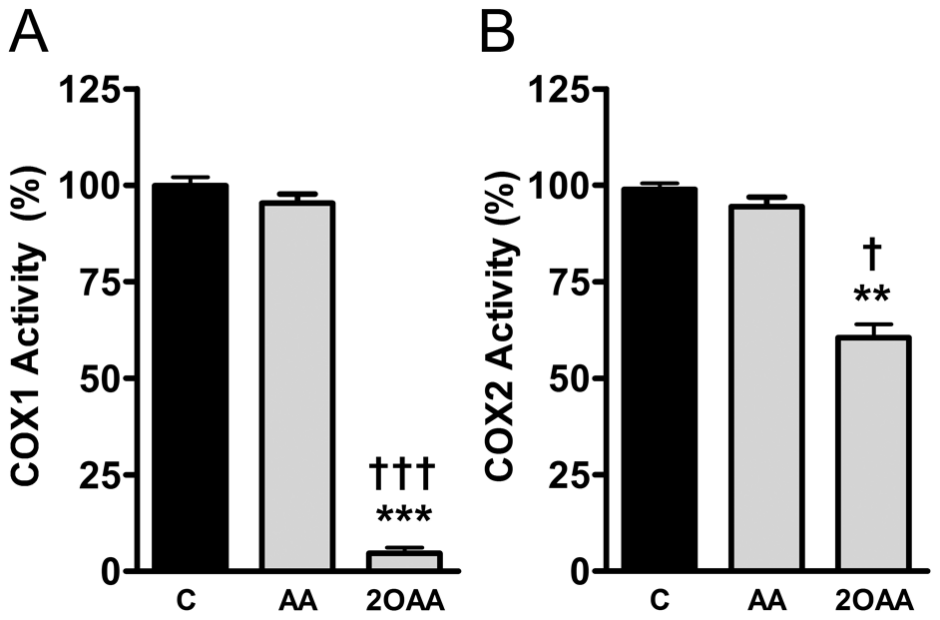
2OAA inhibits COX1 and COX2 activity. Bar diagram showing the inhibition of **A**. COX1 or **B**. COX2 activity determined by measuring PGH_2_ production in the presence of 2OAA (250×10^−6^ M) and AA (250×10^−6^ M), and compared with untreated control cells (100%: ** *p*<0.01, *** *p*<0.001 with respect to controls; † *p*<0.05, ††† *p*<0.001 with respect to AA; n = 6).

We determined the effect of AA and 2OAA on purified COX1 and purified COX2 activity by measuring PGH_2_ production in an *in vitro* quantitative cell-free assay [25]. While 2OAA (250×10^−6^ M) exerted a dramatic inhibitory effect on COX1 activity, and a marked and significant inhibition of COX2 activity (Fig. 3A and 3B), AA did not significantly affect COX1 or COX2 activity at the same concentration (Fig. 3A and 3B).

### 2OAA downregulates COX2 expression in LPS-stimulated U937 cells

**Figure 4:**
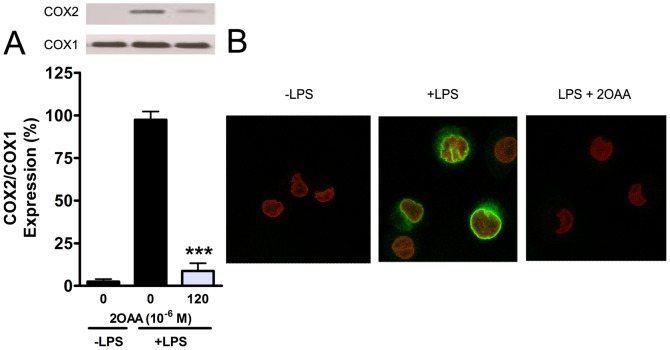
2OAA decreases LPS-induced COX2 protein levels in differentiated human U937 cells challenged with LPS. **A**. A representative immunoblot and bar diagram showing the effect of 2OAA (120×10^−6^ M; 6 h) on COX1/COX2 expression in differentiated U937 cells previously challenged with LPS (62 ng/ml; 6 h). Results are expressed as the COX2/COX1 ratio in untreated cells in the presence of LPS (100%: *** *p*<0.001; n = 6). Expression of the constitutive COX1 isoform was unaffected by either LPS or 2OAA treatment. **B**. Confocal micrographs showing the absence of COX2 (green) expression (left panel) in unchallenged differentiated human U937 cells, the characteristic perinuclear induction of COX2 (green) in LPS-challenged (62 ng/ml; 18 h) differentiated human U937 cells (middle panel), and the inhibition of COX2 expression produced by exposing these cells to 2OAA (120×10^−6^ M; 18 h). Cell nuclei were stained with propidium iodide (red).

To determine the effect of 2OAA administration on the expression of both COX isoforms, differentiated U937 macrophage like cells were challenged with LPS (62 ng/ml; 6 h) in the presence or absence of 2OAA (120×10^−6^ M; 6 h), and COX isoform expression was evaluated in Western blots. As described previously, LPS markedly increased the expression of the inducible COX2 isoform without affecting that of the constitutive COX1 isoform (Fig. 4A) [28]. In LPS-challenged cells, treatment with 2OAA (120×10^−6^ M, 6 h) resulted in a marked reduction in COX2 protein, while those of COX1 remained unchanged (Fig. 4A). To further study the effect of 2OAA on COX2 expression, differentiated U937 cells were challenged for longer with LPS (62 ng/ml; 18 h) in the presence or absence of 2OAA (120×10^−6^ M; 18 h) and they were analyzed by confocal microscopy using an anti-COX2 antibody. After stimulation with LPS, the cells exhibited characteristic perinuclear localization of the induced COX2, although in the presence of 2OAA there was a marked reduction in COX2 expression (Fig. 4B).

**Figure 5:**
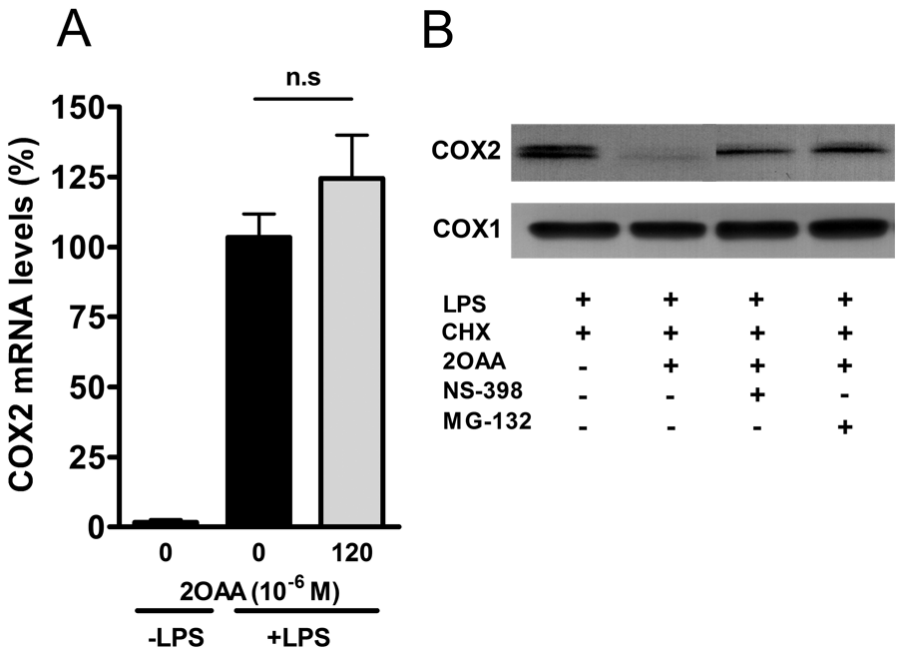
Proteolysis of COX2 by 2OAA. **A**. Bar diagram showing the upregulation of COX2 mRNA in differentiated U937 cells challenged with LPS (62 ng/ml). 2OAA (120×10^−6^ M; 6 h) failed to downregulate COX2 mRNA levels despite its inhibitory effect on COX2 protein. **B**. Representative immunoblot showing COX2 protein in the presence or absence of the general protein synthesis inhibitor CHX (50×10^−6^ M), the chemical blocker of the COX2 active site NS-398 (20×10^−6^ M), and the 26S proteasome inhibitor MG132 (20×10^−6^ M) plus 2OAA (120×10^−6^ M). Constitutive COX1 protein expression is shown as a Western blot loading control.

We also evaluated the effect of 2OAA on COX2 mRNA expression in differentiated U937 macrophages challenged with LPS (62 ng/ml; 6 h). As expected, LPS markedly increased COX2 mRNA expression and despite its inhibitory effect on COX2 protein expression, exposure to 2OAA (120×10^−6^ M, 6 h) failed to downregulate COX2 mRNA, suggesting the existence of a post-translational regulatory mechanism (Fig. 5A). It was previously demonstrated that AA downregulates COX2 expression by inducing the degradation of COX2 protein via two distinct mechanisms: ubiquitination and degradation through the ubiquitin-proteasome system; and less well understood mechanism triggered by the binding of AA to COX2, known as suicide inactivation [29]. To study these proteolytic mechanisms, differentiated U937 macrophage-like cells challenged with LPS (62 ng/ml; 6 h) were treated for 2 h with the ribosome inhibitor CHX (50×10^−6^ M), the selective COX2 inhibitor NS-398 (20×10^−6^ M) or the 26S proteasome inhibitor MG-132 (20×10^−6^ M), both in the presence or absence of 2OAA. LPS markedly induced the expression of the inducible COX2 protein as evident in Western blots (Fig. 5B), whereas treatment with 2OAA (120×10^−6^ M, 2 h) provoked a marked reduction in COX2 protein. When 2OAA (120×10^−6^ M, 2 h) was combined with either NS-398 (20×10^−6^ M) or MG-132 (20×10^−6^ M), COX2 protein expression recovered (Fig. 5B), indicating that both suicide inactivation and proteasome-dependent proteolysis are triggered by 2OAA.

### 2OAA inhibits NO production in LPS-stimulated BV-2 cells

**Figure 6:**
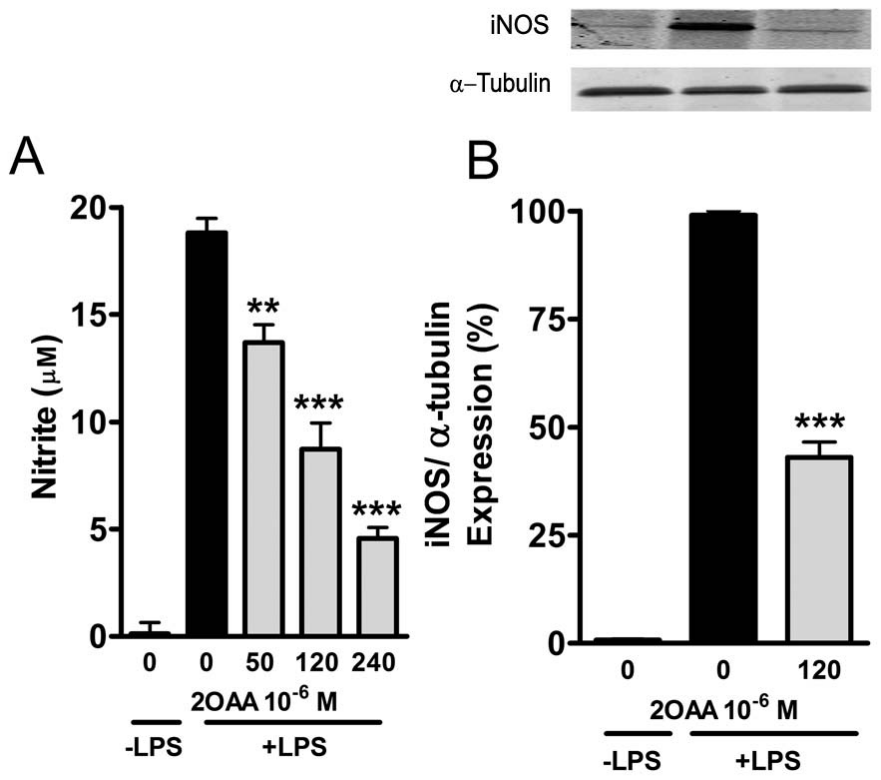
2OAA attenuates the increase in iNOS protein levels and NO production induced by LPS in BV2 murine microglial cells. **A**. Bar diagram showing the dose-dependent effect of 2OAA (50, 120 and 240×10^−6^ M; 24 h) on NO production (measured by the Griess assay: see Materials and Methods) by BV2 mouse microglial cells previously challenged with LPS (1 µg/ml; 24 h). Results are expressed relative to the NO production in untreated cells in the presence of LPS (100%: ** *p*<0.005, *** *p*<0.001; n = 6). **B**. A representative immunoblot and bar diagram showing the effect of 2OAA (120×10^−6^ M; 24 h) on iNOS expression in BV2 mouse microglial cells previously challenged with LPS (1 µg/ml; 24 h). Results are expressed relative to the NO produced by untreated cells maintained in the presence of LPS (100%: *** *p*<0.001; n = 6).

To investigate the effect of 2OAA on the production of NO, BV-2 cells were challenged with LPS (1 µg/ml; 24 h) in the presence or absence of 2OAA at different concentrations (50×10^−6^, 120×10^−6^ and 240×10^−6^ M; 24 h). NO production was assessed by quantifying nitrite accumulation in the culture medium (the final stable product of nitric oxide) using the Griess reagent [26]. As expected, LPS induced a significant increase in NO production, although 2OAA (50×10^−6^, 120×10^−6^ and 240×10^−6^ M) diminished the LPS-induced production of NO in a concentration-dependent manner (Fig. 6A).

### 2OAA downregulates iNOS expression in LPS-stimulated BV-2 cells

We tested the effect of 2OAA on the expression of iNOS in BV-2 cells challenged with LPS (1 µg/ml; 24 h) and in Western blots, it was evident that exposure to LPS alone markedly increased the expression of iNOS ^c^ompared to that seen in unstimulated cells (Fig. 6B). By contrast, the presence of 2OAA (120×10-6 M; 24 h) significantly dampened the expression of iNOS protein induced by LPS stimulation (Fig. 6B).

### 2OAA reduced the serum TNF-α produced in LPS-challenged mice

**Figure 7:**
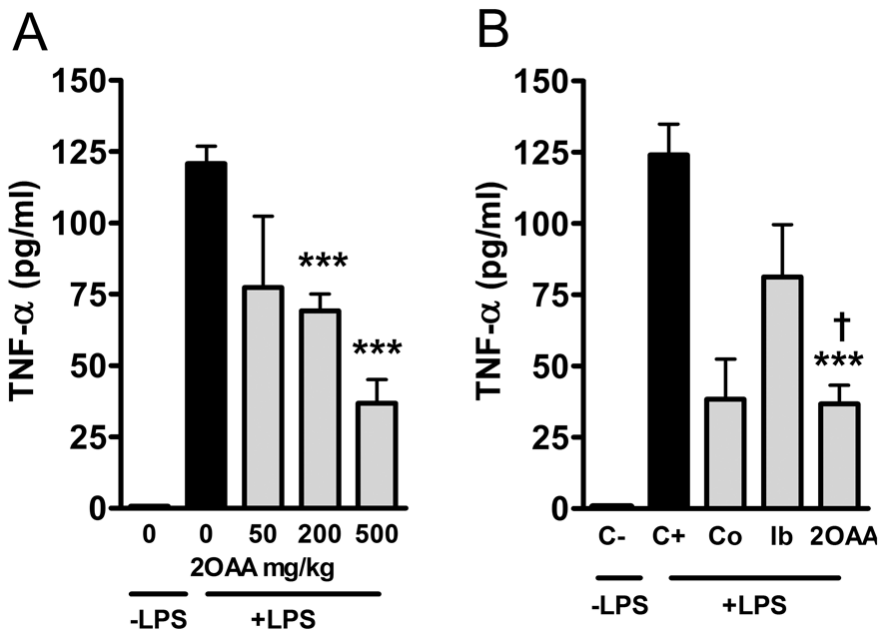
2OAA attenuates the LPS-induced increase in serum TNF-α levels in C57BL6/J mice. **A**. Bar diagram showing the dose-dependent inhibition of serum TNF-α by 2OAA (50, 200 and 500 mg/kg) in LPS-challenged C57BL6/J mice (100%: *** *p*<0.001 with respect to the LPS-challenged controls). **B**. Bar diagram showing the inhibitory effects of cortisone (12.5 mg/kg; Co), ibuprofen (7.5 mg/kg; Ib) and 2OAA (500 mg/kg) on serum TNF-α in LPS-challenged C57BL6/J mice (*** *p*<0.001 with respect to controls; † *p*<0.05 with respect to Ib).

To study the *in vivo* efficacy of 2OAA in a mouse model of inflammation, we orally administered 2OAA (50 to 500 mg/kg) to C57BL6/J mice 90 min before they received a LPS challenge (20 µg/g, i.p.). LPS induced a marked and significant increase in serum TNF-α levels, which was attenuated by 2OAA treatment in a dose-dependent manner (Fig. 7A). This effect of 2OAA *in vivo* was compared with that of cortisone and ibuprofen and we found that the anti-inflammatory effect of 2OAA in mice (reflected in an attenuation of the LPS-induced increase in TNF-α) was significantly greater than that of ibuprofen and similar to that of cortisone, a steroid compound at therapeutic doses (Fig. 7B).

## Discussion

Understanding protein structure and structure-function relationships has provided significant insight into the molecular bases of protein-ligand interactions [30], [31], also forming the basis for the rational design of drugs to treat human diseases with unmet clinical needs [32]. The goal of the present study was to rationally design a nonsteroidal anti-inflammatory drug (NSAID) with similar potency to that of steroid compounds but that lacked the significant side-effects of these drugs. For this purpose, we first tested AA (the main COX substrate) analogues using computer assisted tools, then comparing the potential toxicity and efficacy of 2OAA with that of AA in cellular and animal models. Our molecular docking simulations predicted similar binding energies for 2OAA and AA to COX1, and an enhanced interaction between 2OAA and COX2 than that of AA. This suggests that 2OAA competes with AA to bind to COX isoforms and therefore, that it interferes with their enzymatic activities to inhibit the synthesis of pro-inflammatory mediators. In this context, the development of COX inhibitors represents a landmark in the evolution of NSAIDs [33]. However, AA derivatives are not usually considered potential anti-inflammatory compounds, possibly because their structure is too similar to that of COX substrates and products. In a cell, AA is metabolized by both COX1 and COX2, is converted into pro-inflammatory PGs and TXs [6], [7]. By contrast, we propose that 2OAA is a potent anti-inflammatory compound with low toxicity and acting as a COX inhibitor, probably due to its similarity to the natural compound.

While AA (240×10^−6^ M) compromised microglial BV-2 cell viability by 75% as shown by the MTT and Trypan blue staining methods no toxic effects on cell growth were observed for 2OAA under identical experimental conditions, indicating that the 2-hydroxylation of AA attenuates its toxic effects.

We compared the effect of AA and this non-toxic analogue on purified COX1 or purified COX2 activities by measuring PGH_2_ production in a cell-free system. Although the binding energies predicted by the simulations of molecular docking to COX1 and COX2 were very similar, *in vitro* 2OAA appeared to inhibit COX1 activity more strongly than AA. This might reflect the fact that the binding of a ligand in a catalytically competent orientation is necessary but not sufficient to induce enzymatic catalysis. Among the initial steps in the formation of PGH_2_, a tyrosine is oxidized to a free radical before catalysis can begin. The subsequent abstraction of the AA 13-*pro*-S hydrogen by Tyr-385 results in a pentadienyl radical, centered on C11, C13 and C15. In addition, calculation of the Fukui function indicates that the presence of the hydroxyl group reduces the potential reactivity of 2OAA. This observation is consistent with the longer period over which 2OAA remains at the active site of the COX enzymes and it is in agreement with the inhibition observed.

To study the effect of 2OAA on the expression of the inducible COX2, we used human U937 monocytes differentiated to macrophage-like cells, which were stimulated with LPS to simulate inflammation-derived COX2 overexpression. U937-derived macrophages overexpress COX2 protein after LPS stimulation [28], yet 2OAA administration decreases COX2 expression without affecting constitutive COX1 expression. Thus 2OAA not only inhibits the in vitro activity of both COX1 and COX2 but it also significantly reduces COX2 levels while maintaining COX1 protein expression. Previous studies demonstrated that COX2 protein can be ubiquitinated and degraded by the 26 S proteasome, a process that involves exit of the protein from the ER via the ER-associated degradation system [29]. Another mechanism that appears to be independent of the proteasome system (suicide inactivation) involves the degradation of the enzyme after the binding of its natural substrate [6]. In the present study, we demonstrate that both 26S proteasome inhibition (MG-132) and COX2 blockade at the active site (NS-398) prevent proteolytic degradation of COX2. These results suggest that both the proteasome and suicide inactivation pathways are involved in 2OAA-induced COX2 degradation. This anti-inflammatory mode of action, involving COX2 proteolysis, partially explains the potent effect of 2OAA. While this work is focused on the effect of 2OAA on COXs inhibition, it is conceivable that 2OAA could also have a variety of effects at other different levels. For example, membrane AA could be displaced by 2OAA decreasing the concentration of AA available for PLA2. In this context, 2OAA could also bind and interfere with the activity of PLA2 affecting the release of stored AA which is the rate-limiting step for icosanoid generation [2], [4]. Another possibility not explored in this work, is the possible effect of 2OAA on LOXs activities and expression. Of particular interest would be the study of the effect of 2OAA on 5LOX due to its important role on the synthesis of hydroxyacids and leukotrienes implicated in inflammatory and allergic disorders.

Microglial cells are the resident macrophage-like cells of the central nervous system and they are broadly implicated in neuronal survival, innate immunity, microbial infection and brain damage. Microglial over-activation (excessive production of PGs, superoxide, NO and cytokines) can lead to inflammatory neuropathologies [34]. Since iNOS expression can be inhibited by natural lipids [35] and by modified lipids, such as oleanolic acid-cyano-derivatives [36], we investigated whether 2OAA also inhibited NO synthesis. Stimulation of microglial BV-2 cells with LPS increased NO production and iNOS expression, as described previously [37], yet these effects were attenuated by 2OAA in a dose dependent manner. These results suggest that 2OAA inhibits the production of NO derivatives such as peroxynitrites, minimizing the damage to proteins by oxidation. The AA, COX2 and iNOS pathways are highly interconnected, with NO stimulating both PLA-2 and COX2 activity, regulating AA release, and promoting eicosanoid and PG synthesis [38]. Thus, 2OAA is an anti-inflammatory AA derivative with a dual-mechanism of action, simultaneously targeting excessive NO and PG synthesis. The 2OAA was originally designed to inhibit COX1 and COX2 activities. Our results showed that besides the inhibition of the activity of both COXs isoforms, 2OAA induced COX2 protein degradation and iNOS down-regulation. Therefore, further studies are required to elucidate the cellular pathways and the mechanisms that are affected by this new compound.

Finally we confirmed the *in vivo* efficacy of 2OAA by measuring plasma of TNF-α levels. We selected a transient LPS-induced endotoxemia model in C57BL6/J mice [39]. 2OAA readily reduced the serum TNF-α levels in a dose-dependent manner, producing a stronger effect than that of ibuprofen, similar to that of cortisone at the therapeutic doses used. The FDA indicates that the dose to be used in humans should be those used in mice multiplied by 0.08 [27]. Thus, a dose of 500 mg/kg in mice would correspond to 2.8 grams in humans (calculations made for a weight of 70 kg), a dose that falls within the range of the daily amount of NSAIDs currently prescribed (e.g., Ibuprofen). These findings constitute a proof of relevance, demonstrating greater efficacy of 2OAA than a commonly used NSAID like ibuprofen. Furthermore, its comparable efficiency to cortisone and low toxicity suggest that 2OAA may replace this steroidal anti-inflammatory drug for certain treatments.
